# Examining human paragonimiasis as a differential diagnosis to tuberculosis in The Gambia

**DOI:** 10.1186/s13104-018-3134-y

**Published:** 2018-01-15

**Authors:** Richard Morter, Ifedayo Adetifa, Martin Antonio, Fatima Touray, Bouke C. de Jong, Charlotte M. Gower, Florian Gehre

**Affiliations:** 1 Vaccines and Immunity Theme, Medical Research Council (MRC) Unit The Gambia, Fajara, The Gambia; 2 School of Biological Sciences, The University of Manchester, Manchester, UK; 3 Disease Control and Elimination Theme, Medical Research Council (MRC) Unit The Gambia, Fajara, The Gambia; 4 Department of Infectious Disease Epidemiology, London School of Hygiene and Tropical Medicine, London, UK; 5 Microbiology and Infection Unit, Warwick Medical School, University of Warwick, Coventry, UK; 6 Faculty of Infectious and Tropical Diseases, London School of Hygiene & Tropical Medicine, London, UK; 7 Department of Medicine, New York University, New York, USA; 8 Mycobacteriology Unit, Institute of Tropical Medicine, Antwerp, Belgium; 9 Department of Infectious Disease Epidemiology, Imperial College London, London, UK

**Keywords:** Paragonimiasis, Paragonimus, West Africa, Foodborne trematodiases, Neglected tropical diseases, Tuberculosis

## Abstract

**Objective:**

Paragonimiasis is a foodborne trematode infection of the lungs caused by *Paragonimus* spp., presenting clinically with similar symptoms to active tuberculosis (TB). Worldwide, an estimated 20.7 million people are infected with paragonimiasis, but relatively little epidemiological data exists for Africa. Given a recently reported case, we sought to establish whether paragonimiasis should be considered as an important differential diagnosis for human TB in The Gambia, West Africa.

**Results:**

We developed a novel PCR-based diagnostic test for *Paragonimus* species known to be found in West Africa, which we used to examine archived TB negative sputum samples from a cross-sectional study of volunteers with tuberculosis-like symptoms from communities in the Western coastal region of The Gambia. Based on a “zero patient” design for detection of rare diseases, 300 anonymised AFB smear negative sputum samples, randomly selected from 25 villages, were screened for active paragonimiasis by molecular detection of *Paragonimus* spp. DNA. No parasite DNA was found in any of the sputa of our patient group. Despite the recent case report, we found no evidence of active paragonimiasis infection masking as TB in the Western region of The Gambia.

**Electronic supplementary material:**

The online version of this article (10.1186/s13104-018-3134-y) contains supplementary material, which is available to authorized users.

## Introduction

Tuberculosis (TB) is a major global public health concern. The mainstay of TB diagnosis, particularly in developing countries, is sputum smear-microscopy [1]. As a result, verifying differential diagnosis for persons with clinical symptoms and a smear-negative microscopy result is difficult. Besides smear negative, culture positive TB, common alternative diagnoses include non-tuberculous mycobacteria and in some parts of the world, paragonimiasis, a food-borne trematode infection caused by the *Paragonimus* spp. [2].

*Paragonimus* is a highly neglected and poorly known infection acquired through ingestion of undercooked crustacea [3, 4], which infects a range of mammals including humans, where symptoms are synonymous with TB infection [5, 6]. Diagnostic confusion between TB and paragonimiasis is common in countries such as India [6] and The Philippines [7], and has resulted in investment in training and integrated diagnostic procedures for all TB suspects in some areas. In West Africa, epidemiological data describing the burden of paragonimiasis is scarce, with studies limited to Nigeria and Cameroon [8, 9]. Though case reports from Liberia, Benin and Cote d’Ivoire have also been documented [10–12]. Whilst the species *Paragonimus westermani* and *P. heterotremus* are the most common species worldwide, in Africa only *P. africanus* and *P. uterobilateralis* have been found thus far [12, 13].

In The Gambia, West Africa, there is anecdotal evidence of *Paragonimus* infection and crustaceans are included in the local diet, although there is little documented evidence of culinary practices that might increase risk of consuming undercooked crabs. As recently as 2011, there was a confirmed case in a 12 year old Gambian boy returning from Casamance, the Senegalese region immediately south of our study area, who was infected with *Paragonimus* after reportedly consuming raw crabmeat (Richard Bradbury, personal communication). In addition, four case reports were described in patients originating from Casamance in the 1960s [12, 14]. Therefore, one possible alternative diagnosis for individuals who are smear-negative with TB-like symptoms in The Gambia could be paragonimiasis.

Nested into a cluster-randomised TB enhanced case finding (ECF) study conducted in The Gambia from December 2011 to November 2014, we carried out the first epidemiological study of paragonimiasis amongst smear-negative Gambian TB suspects living in the coastal region bordering Casamance. Using archived sputum samples from the ECF study, our aim was to conduct a rapid appraisal of whether human paragonimiasis was an important differential diagnosis for TB in this region of the Gambia, which might warrant more extensive surveys and investment in training, awareness and diagnostic facilities for this neglected parasite. We further aimed to develop a diagnostic test for paragonimiasis suitable for surveys in West Africa.

## Main text

### Materials and methods

#### Study area

For the TB-ECF study, field teams sensitised community members in villages through video presentations in local languages explaining TB symptoms, diagnosis and treatment. All villages were located in the coastal Greater Banjul area of The Gambia and the area bordering Casamance in southern Senegal. Patients with self-reported coughing and production of two sputum samples were enrolled regardless of age and gender. Written informed consent was provided by all participants including written parental consent for minors and assent where appropriate. No additional exclusion criteria applied to those meeting the criteria for inclusion. For the paragonimus study, a subset of 300 archived patient sputa negative on two Acid-fast Bacteria (AFB) smears were tested for the presence of *Paragonimus* DNA, therefore the investigators were not blinded to AFB-smear status. AFB smear microscopy was performed on fresh samples received directly from the field before sample archiving. Sample size calculation for this study was based on the “zero prevalence” model for detection of rare diseases [15]. This model calculates that an absence of cases found in 300 samples would mean it could be stated with 95% confidence that prevalence of an infection is < 1% amongst the study population of smear-negative TB suspects. In order to maximise geographic coverage, twelve anonymised AFB-smear negative sputum samples originating from each of 25 villages in the ECF study were randomly selected for analysis.

#### Sputum sample processing and DNA extraction

An equivolume of mucolysis solution (0.5% *N*-acetyl l-cysteine, 2% sodium hydroxide, 1.45% tri-sodium citrate) was added to 1.5 ml of stored sputa, vortexed and incubated at room temperature. After 15 min, each sample was made up to a volume of 50 ml with phosphate buffered saline (PBS) and centrifuged at 3000 rpm for 20 min. The pellet was re-suspended in 200 µl PBS and heat inactivated at 99 °C for 20 min.

DNA was purified from 200 µl of each sample using QIAamp DNA mini kit spin columns (Qiagen Ltd., Hilden, Germany, Ref. 51306) following the standard manufacturer’s protocol for extraction from body fluids. The DNA was eluted in a final volume of 150 µl elution buffer and stored at − 20 °C.

Successful DNA extraction was monitored by real-time amplification of the human ribonuclease P gene (RNaseP) as an internal positive control. Reagents were prepared in 25 µl final volume to the following concentrations: 1 × Platinum^®^ Quantitative PCR SuperMix-UDG (Invitrogen, Massachusetts, USA, Ref 1173-025), 0.2 pmol of each primer; RNaseP-F and RNaseP-B, 0.2 pmol probe RNaseP-P, 3.0 mM MgCl_2_, 0.1x ROX reference dye (Invitrogen, Ref. 12223-012) and 2.5 µl template DNA. Primer and probe sequences are found in Additional file 1: Table S1. In an ABI7500 real-time PCR system (Applied Biosystems, Massachusetts, USA), samples were amplified with the following cycling parameters: 1 cycle at 50 °C/2 min, 1 cycle at 95 °C/10 min, followed by 45 cycles of 95 °C/15 s and 60 °C/1 min.

#### *Paragonimus* spp. PCR assay development

The internal transcribed spacer 2 (ITS-2) region of *Paragonimus* spp. was chosen as the target region for the PCR assay. Sequences were obtained from GenBank for *P. africanus* (accession no. AB298780) and kindly provided by Professor David Blair (James Cook University, Australia) for *P. uterobilateralis* (unpublished data). The assay used was modified from a PCR-based assay published by Chen et al. (2011). This assay was designed to target the ITS-2 sequence of *P. westermani* (primers PW-F and PW-B), for our study of paragonimiasis in The Gambia, however, the reverse primer was redesigned for greater specificity to the endemic species; *P. africanus* and *P. uterobilateralis* (PAU-B) (for primer sequences see Additional file 1: Table S1).

Due to the highly neglected status of paragonimiasis in West Africa, no *P. africanus* or *P. uterobilateralis* control worms were available despite efforts to source them. Thus positive control plasmids encoding ITS-2 were synthesised for both *P. africanus* and *P. uterobilateralis* (Eurofins Genomics, Ebersberg, Germany) and amplified in the following PCR conditions: 1U *Taq* PCR Master Mix Kit (Qiagen Ltd., Hilden, Germany, Ref. 201445), 10 pmol of each forward (PW-F) and reverse primer (PW-B or PAU-B), 1 µl template DNA and 9.5 µl PCR-grade H_2_O in a final reaction volume of 25 µl. PCR conditions included an initial denaturation at 94 °C/5 min, 35 cycles 94 °C/30 s (denaturation), 55 °C/1 min (annealing) and at 72 °C/30 s (extension) and a final extension at 72 °C/5 min. The expected PCR product size was 221 base pairs (bp).

## Results

### Study population

Descriptive patient characteristics of 300 smear-negative patients from an enhanced case-finding study for TB in the Greater Banjul region of The Gambia whose sputum samples were randomly selected for inclusion in this study are shown in Table 1.

**Table 1:** Descriptive patient characteristics

	N (%)
Gender	
Female	171 (57)
Male	129 (43)
Age	
< 10	9 (3)
10–25	57 (19)
26–40	75 (25)
41–55	89 (29.7)
56–70	47 (15.7)
> 71	23 (7.7)

### *Paragonimus* spp. PCR assay validation

**Fig. 1:**
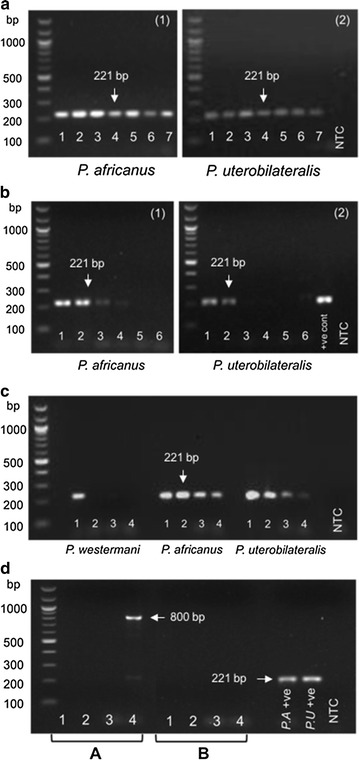
Assay validation electrophoresis gels. **a** To estimate the assay’s limit of detection of pure DNA, PCR was performed on a dilution series of plasmid positive control (1000, 100, 10, 5.0, 1.0, 0.5 and 0.1 pg/µl). Amplification was seen in all dilutions for *P. africanus* and *P. uterobilateralis*. Dilutions ranged from 1.0 ng/µl (dilution 1) to 0.1 pg/µl (dilution 7). Samples were run on the same gel with *P. africanus* and *P. uterobilateralis* samples, and a no template control (“NTC”). **b** To estimate the assay’s limit of detection of DNA in sputum, PCR was performed using DNA extracted from sputum spiked with positive control DNA. 12 sputum concentrates were spiked using the same dilution series as in (**a**). Samples were run on the same gel, with *P. africanus* and *P. uterobilateralis* samples, with plasmid positive control (“+ve cont”) and no template control (“NTC”). **c** Sensitivity of the PW-F/PAU-B primer set to the two West African target *Paragonimus* species was compared to *P. westermani* in a dilution series of pure plasmid positive control (1000, 100, 10 and 5.0 pg/µl). **d** Specificity of PW-F/PAU-B (Series A) designed for *P. africanus* and *P. uterobilateralis* were compared to that of primers PW-F/PW-B (Series B) for *P. westermani*. DNA from known positive patient samples infected with related worm species to *Paragonimus* were tested, including, (1) *Schistosoma mansoni*, (2) *Schistosoma haematobium*, (3) *Necator americanus*, (4) *Opisthorchis* spp. Amplification of a large sized product (800 base pairs) was seen for *Opisthorchis* spp. using the *P. africanus/P. uterobilateralis* primers. No unspecific amplification was seen using primers PW-F/PW-B

The modified assay’s (PW-F/PAU-B) detection limit was estimated using a dilution series of the plasmid positive control DNA. Dilutions of the *P. africanus* and *P. uterobilateralis* positive controls were prepared to the following concentrations: 1000, 100, 10, 5, 1, 0.5, 0.1 pg/µl. 1 µl of each was used as template DNA per reaction using primers PW-F/PAU-B. Overall the PCR assay demonstrated sensitivity at levels lower than 0.1 pg/µl when pure positive control alone was used as template DNA (Fig. 1a). The same dilution series of positive control DNA was then used to spike 200 µl decontaminated sputum sediment. Extracted sputum samples, spiked with plasmid positive control displayed sensitivity levels of 5 and 100 pg/µl of DNA for *P. africanus* and *P. uterobilateralis,* respectively (Fig. 1b). The sensitivity of the PW-F/PAU-B assay was also shown to be greater for the two West African target species, *P. africanus* and *P. uterobilateralis*, compared to *P. westermani* (Fig. 1c).

Primer specificity was validated by assaying known positive DNA of helminth species closely related to *Paragonimus* spp. using both PW-F/PW-B and our adapted PW-F/PAU-B primer combinations (Fig. 1d). The *Paragonimus* spp. samples produced a band of 221 bp as expected. No amplification was detected with either primer pair and *S. mansoni, S. haematobium* or *N. americanus* DNA. There was no amplification product using *Opisthorchis* DNA and the primer combination PW-F/PW-B. Amplifying *Opisthorchis* DNA using our modified primers PW-F/PAU-B resulted in a substantially larger band (800 bp), clearly distinguishable from *Paragonimus* bands (221 bp).

### PCR analysis of clinical samples

The quality of the DNA extractions was verified in 10% (*n* = 30) of samples, selected at random. All samples were positive for RNaseP, suggesting DNA was successfully extracted from sputa (data not shown). We selected the PW-F/PAU-B combination for analysis of the clinical samples. All TB smear-negative sputum samples (*n* = 300) were tested. 10.7% (*n* = 32/300) samples amplified a band larger than the expected product size, approximately 250 bp. Twenty of these potentially positives were validated by DNA sequencing (Macrogen, Amsterdam, The Netherlands), analysed using Geneous V.7.1 sequence alignment and editing software (Biomatters, Auckland, New Zealand) and it was confirmed none of these amplicons were *Paragonimus* spp. sequences through BLAST searches against the GenBank (NCBI, MD, USA) database. These results indicate that no *Paragonimus* infection was detected in the sputa examined in this study. Based on the zero prevalence model, a case detection rate of zero was interpreted (with 95% confidence) as a prevalence less than 1% in our study population.

## Discussion

Paragonimiasis is a neglected yet emerging global health problem. It is especially important as differential diagnosis of TB [6, 16] and its potential large cost implications of mis-diagnosis for TB control programs. Although previous reports suggest that the disease is present in the Senegambian region we did not detect any cases of *Paragonimus* DNA in samples from symptomatic but TB-smear negative patients. The sample size was based on the recommended zero patient design [15]. We used a prevalence of 1% as this is the criterion often used to define parasitic diseases of public health importance [17]. Our findings are in accordance with recent studies in Côte d’Ivoire, West Africa, which also found an absence of active cases of infection, amongst people attending an anti-tuberculosis centre, despite a 25% prevalence of anti-Paragonimus antibodies [11] and the presence of infective metacercariae in approximately 12% of local crabs collected [18]. Overall, our study established that paragonimiasis, although possibly still present in our study population at a low prevalence, is not common enough to be considered a primary differential diagnosis for adults suspected to have TB in this region of the Gambia. However, it must be remembered that sub-population variation in eating practices may alter the risk in children or in other parts of the Gambia. Further work is needed to identify the source of symptoms in smear negative TB suspects.

## Limitations

- Our results are not representative of the whole country although 80% of all TB cases in the country occur in the Greater Banjul area [19].
- We did not study intermediate hosts (crustacea/shellfish) or their mammalian predators and thus our conclusions are limited to the importance of paragonimiasis as a differential diagnosis for TB in this study population, rather than for the generalised presence of paragonimus in other mammals and the potential for occasional human infections.
- This study used archived samples in order to conduct a rapid assessment. Thus we were not able to directly ask participants about individual level eating habits. Blood was not available that would have allowed testing for anti-paragonimus antibodies. Positive control DNA from adult worms was not available.

## Additional file

**Additional file 1: Table S1.** Primer and probe sequences used in the study.

## Abbreviations

TB: tuberculosis
ECF: enhanced-case-finding
AFB: acid-fast bacteria
DNA: deoxyribonucleic acid
PBS: phosphate buffered saline
PCR: polymerase chain reaction
RNaseP: human ribonuclease P
ITS-2: internal transcribed spacer 2
NTC: no template control
bp: base pairs
