# Supplementary material for: Examining human paragonimiasis as a differential diagnosis to tuberculosis in The Gambia
Source: BMC Res Notes. 2018 Jan 15;11:31. doi: 10.1186/s13104-018-3134-y (PMC5769439; doi:10.1186/s13104-018-3134-y)
Supplement: Supplementary file 1 — Additional file 1: Table S1. Primer and probe sequences used in the study. [file 13104_2018_3134_MOESM1_ESM.docx]

**Additional Table S1: Oligonucleotide primer and probe sequences.**

| Assay | Primer/probe | Oligonucleotide sequence |
| --- | --- | --- |
| *Paragonimus* detection | PW-F  (Forward) | 5’-CAA AAA GTC GCG GCT TGG-3’ |
|  | PW-B  (Reverse) | 5’-CAC GCG CAA CAT GAA CCA-3’ |
|  | PAU-B  (Reverse) | 5’-CAC GCG CAG CAT CAA CCA-3’ |
| RNaseP | RNaseP-F  (Forward) | 5’-AGA TTT GGA CCT GCG AGC G-3’ |
|  | RNaseP-B  (Reverse) | 5’-GAG CGG CTG TCT CCA CAA GT-3 |
|  | RNaseP-P  (Probe) | 5’-FAM-TTC TGA CCT GAA GGC TCT GCG CG-BHQ-3’ |
